# Lung cancer in the Swiss HIV Cohort Study: role of smoking, immunodeficiency and pulmonary infection

**DOI:** 10.1038/bjc.2011.558

**Published:** 2012-01-12

**Authors:** G M Clifford, M Lise, S Franceschi, M Egger, C Bouchardy, D Korol, F Levi, S Ess, G Jundt, G Wandeler, J Fehr, P Schmid, M Battegay, E Bernasconi, M Cavassini, A Calmy, O Keiser, F Schöni-Affolter, the Swiss HIV Cohort Study

**Affiliations:** 1 International Agency for Research on Cancer, 150 cours Albert Thomas, 69372 Lyon Cedex 08, France; 2 Epidemiology and Biostatistics Unit, The Scientific Directorate, National Cancer Institute, Aviano, Via Franco Gallini 2, 33081 Aviano, Italy; 3 Institute of Social and Preventive Medicine (ISPM), University of Bern, Finkenhubelweg 1, 3012 Bern, Switzerland; 4 Cancer Registry of the Canton of Geneva, 55 Boulevard de la Cluse, 1205 Geneva, Switzerland; 5 Cancer Registry of the Canton of Zurich, Vogelsangstr. 10, 8091 Zurich, Switzerland; 6 Cancer Registry of the Canton of Vaud, CHUV Falaises 1, 1011 Lausanne, Switzerland; 7 Cancer Registry of Basel, Schönbeinstr. 40, 4003 Basel, Switzerland; 8 Cancer Registry of St Gallen and Appenzell, Flurhofstr. 7, 9000 St Gallen, Switzerland; 9 University Hospital, University of Bern, Inselspital PKT2B, 3010 Bern, Switzerland; 10 University Hospital Zurich, University of Zurich, Rämistrasse 100, 8091 Zurich, Switzerland; 11 Cantonal Hospital, Rorschacher Strasse 95, 9007 St Gallen, Switzerland; 12 University Hospital Basel, Petersgraben 4, 4031 Basel, Switzerland; 13 Ospedale Regionale, Via Tesserete 46, Lugano, Switzerland; 14 University Hospital Lausanne, Rue du Bugnon 4, 1011 Lausanne, Switzerland; 15 University Hospital Geneva, Rue Micheli-Du-Crest 24, 1205 Geneva, Switzerland; 16 Coordination and Data Center, Swiss HIV Cohort Study, Mont-Paisible 16, CHUV, 1011 Lausanne, Switzerland

**Keywords:** lung cancer, HIV, AIDS, immunodeficiency, smoking, case–control study

## Abstract

**Background:**

Immunodeficiency and AIDS-related pulmonary infections have been suggested as independent causes of lung cancer among HIV-infected persons, in addition to smoking.

**Methods:**

A total of 68 lung cancers were identified in the Swiss HIV Cohort Study (SHCS) or through linkage with Swiss Cancer Registries (1985–2010), and were individually matched to 337 controls by centre, gender, HIV-transmission category, age and calendar period. Odds ratios (ORs) were estimated by conditional logistic regression.

**Results:**

Overall, 96.2% of lung cancers and 72.9% of controls were ever smokers, confirming the high prevalence of smoking and its strong association with lung cancer (OR for current *vs* never=14.4, 95% confidence interval (95% CI): 3.36–62.1). No significant associations were observed between CD4+ cell count and lung cancer, neither when measured within 1 year (OR for <200 *vs* ⩾500=1.21, 95% CI: 0.49–2.96) nor further back in time, before lung cancer diagnosis. Combined antiretroviral therapy was not significantly associated with lung cancer (OR for ever *vs* never=0.67, 95% CI: 0.29–1.52), and nor was a history of AIDS with (OR=0.49, 95% CI: 0.19–1.28) or without (OR=0.53, 95% CI: 0.24–1.18) pulmonary involvement.

**Conclusion:**

Lung cancer in the SHCS does not seem to be clearly associated with immunodeficiency or AIDS-related pulmonary disease, but seems to be attributable to heavy smoking.

Lung cancer is one of the most common non-AIDS-defining cancers to occur among HIV-infected persons (Dal Maso *et al*, 2009; Franceschi *et al*, 2010; Shiels *et al*, 2011), and shows two- to seven-fold excess risks in comparison with the general population (Grulich *et al*, 2007; Shiels *et al*, 2009; Franceschi *et al*, 2010). In the Swiss HIV Cohort Study (SHCS) subjects, lung cancer risk is three times that in the general Swiss population (Franceschi *et al*, 2010). A large part of this excess can be explained by the high proportion of heavy smokers among HIV-infected persons (Giordano and Kramer, 2005), particularly among intravenous drug users (IDUs) (Clifford *et al*, 2005; Dal Maso *et al*, 2009).

However, HIV infection has been suggested to be associated with increased lung cancer incidence even after controlling for individually collected (Shiels *et al*, 2010) or hypothetically modelled (Engels *et al*, 2006; Chaturvedi *et al*, 2007) smoking history data. Further suggesting a link between lung cancer and HIV-related immunodeficiency, large cohorts of HIV-infected persons (Patel *et al*, 2008; Guiguet *et al*, 2009; Reekie *et al*, 2010) have recently reported strong associations between declining CD4+ counts and lung cancer risk. Nevertheless, other record linkage studies of cancer registries with cohorts of HIV-infected persons (Clifford *et al*, 2005) and/or AIDS registries (Chaturvedi *et al*, 2007; Polesel *et al*, 2010) have failed to observe any link between CD4+ cell counts and lung cancer risk, or any change in risk between the pre-combined antiretroviral therapy (cART) and cART era.

Although HIV is not considered to have any direct carcinogenic effects (Bouvard *et al*, 2009), it has been hypothesised that HIV-associated inflammation in the lungs might predispose to smoking-related lung damage (Engels *et al*, 2006), and lung cancer in HIV-infected persons has been associated with a history of AIDS-related pulmonary diseases (Kirk *et al*, 2007; Shebl *et al*, 2010), which are themselves related to immunodeficiency.

Our aim was to disentangle the independent effects of smoking, HIV-related immunodeficiency and AIDS-related pulmonary diseases on lung cancer among HIV-infected persons in Switzerland.

## Materials and methods

The SHCS is an ongoing study that has been enrolling HIV-infected persons since 1984 from seven large hospitals in Switzerland (http://www.shcs.ch) (Swiss HIV Cohort Study *et al*, 2010), including 103 000 person-years (py) of follow-up until December 2009. Women contribute 31% of py and representation of HIV-transmission categories are balanced between men having sex with men (MSM), IDU and heterosexual/other (35, 29 and 36% of py, respectively). Detailed information on all AIDS-related disease, CD4+ cell count and HIV-related treatments are collected at the time of enrolment, and at each 6-month follow-up visit. Detailed information on smoking history (in number of pack-years) has been routinely collected from all HIV-infected persons under active follow-up in the SHCS since April 2000, for whom current smoking intensity, as well as number of cigarettes smoked per day, is also recorded at each 6-month follow-up visit.

A total of 107 lung cancer cases were identified in SHCS participants, of which 84 were identified from the SHCS database, and 23 additional cases were identified through record linkage with 8 Swiss Cantonal Cancer Registries (Franceschi *et al*, 2010). In all, 10 patients with Kaposi sarcoma (KS) and 13 with lymphoma localised in the lung were excluded. In addition, 6 prevalent cases occurring before, or within 1 month of, SHCS enrolment and 10 diagnosed >6 months after the last SHCS follow-up date were excluded, leaving 68 eligible incident cases occurring during active SHCS follow-up (median follow-up from SHCS enrolment to lung cancer diagnosis=7 years; interquartile range, 3–12 years). Confirmation of histological subtype was available for 65 (94%) of the 68 cases, including 21 adenocarcinoma (International Classification of Disease in Oncology morphology codes (ICD-O) codes 81403; 82523; 82533; 84813), 15 large cell carcinoma (80103; 80123; 80203; 80313; 80823), 14 squamous cell carcinoma (80703; 80713; 80723), 8 small cell carcinoma (80413) and 7 other specified carcinoma (80463; 84303).

For each lung cancer case, five control subjects were matched at random from eligible SHCS participants without lung cancer. Eligible controls had at least the same length of follow-up as did matched cases. Matching criteria were: (1) SHCS centre; (2) gender; (3) HIV-transmission category (IDU, MSM, heterosexual/other); (4) age at enrolment (as close as possible, up to a maximum of 9 years difference); (5) year at reference date (as close as possible, but within the following calendar periods: 1985–1991; 1992–1996; 1997–April 2000; May 2000–2010). April 2000 was included as a key date to match lung cancer cases and controls with respect to the beginning of availability of smoking information. For 2 cases, only 3 and 4 controls, respectively, could be matched, leaving 337 control subjects for this study (Table 1).

Markers of immunodeficiency (CD4+ cell count; CD8+ cell count; CD4+/CD8+ ratio; HIV viral load) were extracted from the SHCS database at two time periods (1–2 years and <1 year) before the reference date, defined for cases as the date of lung cancer diagnosis, and for controls as that occurring after a similar length of SHCS follow-up (to the exact day) as matched cases before lung cancer. We additionally extracted CD4+ cell counts at 2–3, 3–4, 4–5, 5–6, 6–7, 7–8, 8–9 and 9–10 years before the reference date and calculated mean CD4+ cell counts restricted to cases and controls who (1) were under active follow-up and (2) had a valid CD4+ cell count, in each time period. If more than one measurement for any marker of immunodeficiency was available during any one time period, that closest to the reference date was used. Matching was not retained in the long-term comparison and numbers of cases and controls obviously decreased substantially as follow-up went back in time. The nadir CD4+ cell count, defined as the lowest ever reported CD4+ cell count while under active SHCS follow-up, was also extracted for each subject.

Here, cART use was defined as the prescription of at least three antiretroviral drugs, including a protease inhibitor or a non-nucleoside reverse transcriptase inhibitor or three nucleosides, including abacavir. Only persons who had used cART for >1 month before the reference date were classified as users.

The definition of AIDS-related pulmonary disease includes recurrent bacterial pneumonia, pulmonary tuberculosis (TB), or *Pneumocystis jiroveci* pneumonia, recorded at any time before the reference date.

This study was approved by the local ethical committees of the seven SHCS sites and of the International Agency for Research on Cancer. Written informed consent was obtained from all SHCS participants.

### Statistical analysis

Logistic regression, conditioned on matching variables, was used to calculate odds ratios (ORs) and corresponding 95% confidence intervals (95% CIs). Models were also adjusted for smoking status (never/former, current with <30 pack-years, current with ⩾30 pack-years, unknown).

## Results

Table 1 shows the distribution of the 68 lung cancer cases and controls by matching variables. A majority of lung cancer cases were male (79.4%), had been followed up in the SHCS for >5 years before lung cancer diagnosis (64.7%) and were diagnosed after 1996 (86.8%). Intravenous drug users accounted for 36.8% of cases. As a result of matching on these criteria, these proportions were similar among controls. Lung cancer occurred at a mean age of 50 years. Of the 64 lung cancer cases with follow-up post-cancer, only 9 (14%) were still alive at 2 years after lung cancer diagnosis.

The associations of smoking, cART use, AIDS and nadir CD4+ cell count with lung cancer risk are shown in Table 2. Smoking status was known for 52 lung cancer cases (76.5%) and 262 controls (77.7%) (i.e., those followed up in the SHCS after April 2000), of whom 96.2% (11.5% former, 84.6% current) of lung cancer cases and 72.9% (24.0% former, 48.9% current) of controls were smokers. Among controls, the prevalence of smoking was 96.0, 60.0 and 66.4% among IDU, MSM and heterosexual/others, respectively. Lung cancer risk was very strongly associated with current smoking (OR *vs* never=14.4, 95% CI: 3.36–62.1), and was also elevated, although not significantly so, among the few former smokers (OR *vs* never=3.22, 95% CI: 0.63–16.6). Former smokers were at significantly lower risk than current smokers (OR=0.22, 95% CI: 0.08–0.59). Odds ratios were slightly higher among current smokers who had smoked ⩾30 (OR *vs* never=15.9, 95% CI: 3.67–69.1) than <30 (OR *vs* never=11.5, 95% CI: 2.42–54.6) pack-years. History of cART use was not significantly associated with lung cancer risk (OR for ever *vs* never=0.67, 95% CI: 0.29–1.52). History of AIDS-related diseases, whether with pulmonary disease (OR=0.49, 95% CI: 0.19–1.28) or without pulmonary disease (OR=0.53, 95% CI: 0.24–1.18), was not more frequent among cases than controls. Three, three and zero cases of recurrent bacterial pneumonia, *P. jiroveci* pneumonia and TB, respectively, were previously diagnosed among lung cancer cases. No significant associations or trends with lung cancer were observed for nadir CD4+ cell count (OR for <50 *vs* ⩾200=0.73, 95% CI: 0.34–1.55).

The associations of various measures of immunodeficiency with lung cancer risk are shown in Table 3, measured at two different time periods with respect to lung cancer diagnosis (within 1 year and 1–2 years before), and according to two statistical models (unadjusted and adjusted for smoking). No significant associations were observed between lung cancer and CD4+ cell counts, neither when measured within 1 year (OR for <200 *vs* ⩾500=1.21, 95% CI: 0.49–2.96) or 1–2 years (OR=0.96, 95% CI: 0.41–2.24) before lung cancer diagnosis. Similarly, no significant associations or trends with lung cancer were observed for CD8+ cell counts (Table 3). A CD4+/CD8+ ratio lower than 25 within 1 year of lung cancer diagnosis showed an association with lung cancer risk of borderline statistical significance (OR=2.15, 95% CI: 1.00–4.59), but this relationship was not seen at 1–2 years before lung cancer diagnosis (OR=1.07, 95% CI: 0.49–2.36). Although data on HIV viral load were available for a smaller number of cases (*n*=54, 79%) and controls (*n*=269, 80%), no evidence of an association of lung cancer with higher viral load was observed within 1 year of lung cancer diagnosis (OR for ⩾10,000 *vs* <500=1.10, 95% CI: 0.44–2.75).

Adjustment for smoking had no material effect on any of the above findings (Table 3), nor did a sensitivity analysis excluding subjects of unknown smoking status (e.g., smoking adjusted OR for <200 *vs* ⩾500 CD4+ cell counts at 1–2 years before lung cancer=0.93, 95% CI: 0.31–2.76). Among controls, there were no significant correlations between any of the markers of immunodeficiency with smoking status (data not shown).

**Figure 1:**
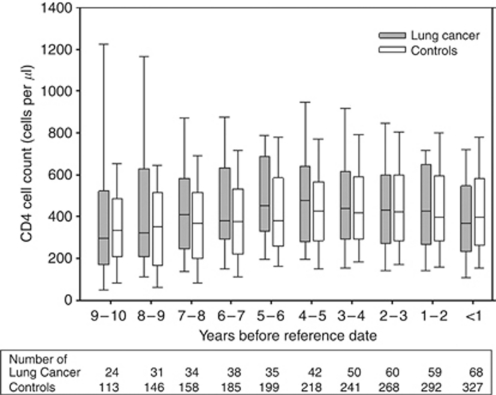
Box plots^a^ of CD4+ cell counts at yearly intervals prior to reference date^b^, among lung cancer cases and controls. ^a^Horizontal lines in box plots represent 10th, 25th, 50th (median), 75th and 90th (percentiles). ^b^See Materials and Methods section for definition of reference date.

Figure 1 shows mean CD4+ cell counts from 10 years to <1 year before the reference date in lung cancer cases and controls. There was no evidence of any difference in CD4+ cell counts between cases and controls in any time period before the reference date.

## Discussion

Our carefully matched case–control study within the SHCS suggests no evidence for a significant effect of HIV-related immunodeficiency on lung cancer risk in this high-risk population (Franceschi *et al*, 2010). None of the classic markers of HIV-related immunodeficiency, including low CD4+ cell counts, high HIV viral load nor history of AIDS or AIDS-related pulmonary disease, showed any clear association with lung cancer in the SHCS.

A strong relationship between declining CD4+ cell counts and lung cancer risk was recently reported by the French Hospital HIV Database (FHVD) (Guiguet *et al*, 2009), with a relative risk of 4.8 (95% CI: 2.8–8.0) for 100–199 *vs* >500 latest CD4+. Similarly strong relationships with CD4+ cell counts have been reported in two additional studies from the United States and Europe (Patel *et al*, 2008; Reekie *et al*, 2010). Although the CIs around our risk estimates are not entirely incompatible with those of previous studies, the findings from the SHCS show no or little association. The reasons for these inconsistencies are unclear, but not all previous studies were supplemented by data linkage with cancer registries to the same extent of the SHCS. Indeed, the FHVD has since been estimated to be only 67% complete with respect to lung cancer diagnosis (Lanoy *et al*, 2011). Thus, in HIV cohorts that do not obtain comprehensive cancer ascertainment, the more immunosuppressed patients may be overrepresented among lung cancer cases as a consequence of investigations of AIDS-related pulmonary diseases. Alternatively, the inability to completely rule out KS and lymphoma localised in the lung, which contributed up to 50% of lung neoplasms in the pre-cART era (Parker *et al*, 1998), could lead to erroneous interpretations, given the strong associations of these two AIDS-defining cancers with declining CD4+ cell counts. In this study, histological verification allowed the exclusion of 10 lung KS and 13 lung lymphomas. Otherwise, the distribution of histological types were similar to those reported in other series of HIV-positive lung cancer, as well as in age-matched series of HIV-negative cases (Lavole *et al*, 2006).

Although confirmed as a very strong risk factor for lung cancer (16-fold increase in risk for ⩾30 pack-years), smoking behaviour was not statistically related to markers of immunodeficiency in the SHCS and hence would appear not to be a strong confounder of the association between these markers and lung cancer. However, confounding by smoking behaviour is more problematic when comparing lung cancer between HIV-positive and HIV-negative subjects (Engels *et al*, 2006; Chaturvedi *et al*, 2007; Shiels *et al*, 2010; Engsig *et al*, 2011). In the face of such a strong relationship, even with adjustment for smoking measures at an individual level (Levine *et al*, 2010; Shiels *et al*, 2010), there remains potential for residual confounding through unmeasured differences in the patterns (e.g., duration of the habit and time since quitting among former smokers) of tobacco use between HIV-positive and HIV-negative ever smokers. A two-fold excess risk of lung cancer is also consistently seen among immunosuppressed kidney transplant recipients (Grulich *et al*, 2007; van Leeuwen *et al*, 2010), which might suggest a role of immunodeficiency. However, smoking is also associated with indications for kidney transplant, so that confounding by smoking history is also difficult to rule out in this scenario (Wen *et al*, 2008).

History of AIDS-related pulmonary disease, and in particular recurrent pneumonia, was recently linked to an increase in lung cancer risk in the large HIV/AIDS Cancer Match study (Shebl *et al*, 2010), suggesting that HIV-related chronic inflammation might potentiate the carcinogenic effects of smoking in the lung (Engels *et al*, 2006). However, this study had to use hypothetical scenarios to address the problem of confounding by smoking behaviour (Shebl *et al*, 2010), which is a risk factor for both pulmonary infections (notably TB (Lin *et al*, 2007; Gajalakshmi and Peto, 2009)) and lung cancer. Although our sample size was much smaller, we were unable to reproduce evidence of such an effect in the SHCS, where only 8.8% of lung cancer cases had a history of AIDS-related pulmonary disease (and none with TB). This proportion was actually slightly lower than among matched controls, as was the proportion of patients with a history of AIDS.

In agreement with the lack of association with CD4+ cell counts and history of AIDS, there was no evidence for an effect of cART use on lung cancer in the SHCS. Although other studies have suggested that lung cancer incidence is increasing in the era of cART (Bower *et al*, 2003), this phenomenon may be largely an artefact of the increased survival of HIV-infected persons and the inability to fully adjust for ageing and corresponding exponential increase in lung cancer by age (Franceschi *et al*, 2010). Indeed, other studies have suggested that the age-standardised incidence of lung cancer is decreasing over time in persons infected with HIV (Silverberg *et al*, 2009; Shiels *et al*, 2011).

If confirmed, the lack of an effect of immunodeficiency on lung cancer risk would not lend support to a role of infection in lung cancer aetiology. Although infection with human papillomavirus has been suggested to have a role in lung cancer, recent large studies in non-HIV infected persons have provided evidence against this hypothesis, particularly among smokers (Simen-Kapeu *et al*, 2010; Koshiol *et al*, 2011).

The SHCS has many strengths, including the duration and regularity of follow-up and comprehensiveness of clinical and laboratory information. Approximately half of HIV-infected persons in Switzerland have been enrolled in the SHCS, and both genders and different risk categories are well represented. The supplementation of cancer diagnoses through linkage with cancer registries (Clifford *et al*, 2005) meant a more comprehensive registration of lung cancer, and the availability of histological and/or cytological confirmation for a majority of cases. The use of a nested case–control approach allowed careful matching for many important correlates of lung cancer risk, smoking and immune status. The principal weakness of the study is the relative small number of lung cancer cases that have accrued in the SHCS, which limits the extent to which small effects of HIV-related immunodeficiency can be ruled out.

As repeatedly noted in HIV-infected cohorts, we observed a high prevalence of smoking in the SHCS (73% among matched controls), and the expected large increased risks for lung cancer among smokers. However, an important finding of this study was the confirmation that, although the lung cancer risk for former smokers did not disappear, it was considerably less than that among current smokers, as seen previously in a cohort of HIV-infected females (Levine *et al*, 2010) and a number of large studies in the general population (IARC, 2007). Thus, the beneficial effects of quitting smoking appear, in relative terms, as important in HIV-infected persons as in the general population (Peto *et al*, 2000), although more important in absolute terms on account of their heavy burden of lung cancer.

As HIV-infected persons live longer in the era of cART, it can be expected that smoking will increasingly manifest its long-term oncogenic potential and that this lethal cancer becomes an increasingly important cause of death. Focusing on ways to help to quit smoking (Elzi *et al*, 2006) would be effective in reducing lung cancer in this high-risk population.

## Figures and Tables

**Table 1:** Distribution of 68 lung cancer cases and 337 controls, according to matching variables

	**Lung cancer**		**Controls**	
	***N***	**(%)**	***N***	**(%)**
*Centre*				
Basel	11	16.2	55	16.3
Bern	9	13.2	42	12.5
Geneva	15	22.1	75	22.3
St Gallen	7	10.3	35	10.4
Vaud	13	19.1	65	19.3
Zurich	13	19.1	65	19.3
				
*Gender*				
Male	54	79.4	270	80.1
Female	14	20.6	67	19.9
				
*HIV-transmission category*				
MSM	19	27.9	95	28.2
IDU	25	36.8	125	37.1
Het/other	24	35.3	117	34.7
				
*Age at lung cancer*^a^ *(years)*				
25–44	24	35.3	132	39.2
45–54	26	38.2	122	36.2
55–64	13	19.1	61	18.1
⩾65	5	7.4	22	6.5
				
*Duration of follow-up before lung cancer*^a^ *(months)*				
<24	11	16.2	55	16.3
24–59	13	19.1	65	19.3
⩾60	44	64.7	217	64.4
				
*Calendar period at lung cancer* a				
1985–1991	3	4.4	15	4.5
1992–1996	6	8.8	30	8.9
1997–April 2000	6	8.8	31	9.2
May 2000^b^–2005	28	41.2	146	43.3
2006–2010	25	36.8	115	34.1

Abbreviations: Het=heterosexual; IDU=intravenous drug user; MSM=men having sex with men.

a Or reference date for controls (date after a similar length of follow-up in the SHCS as matched cases).

b Truncation in mid-2000 to match for availability of smoking information.

**Table 2:** Relative risk for lung cancer by selected characteristics at reference date^a^

	**Lung cancer**		**Controls**			
	***N***	**%**	***N***	**%**	**OR^b^ (95% CI)**	**Smoking-adjusted OR^c^ (95% CI)**
Overall	68		337			
						
*Smoking status*						
Never	2	3.8	71	27.1	1	
Former	6	11.5	63	24.0	3.22 (0.63–16.6)	
Current	44	84.6	128	48.9	14.4 (3.36–62.1)	
Unknown	16		75			
Pack-years^d^						
<30	16	36.4	62	50.8	11.5 (2.42–54.6)	
⩾30	28	63.6	60	49.2	15.9 (3.67–69.1)	
Unknown	0		6			
						
*History of cART use*						
Never	18	26.5	77	22.8	1	1
Ever	50	73.5	260	77.2	0.67 (0.29–1.52)	0.73 (0.31–1.70)
						
*History of AIDS-defining disease*						
No	54	79.4	229	68.0	1	1
Yes, without pulmonary disease^e^	8	11.8	62	18.4	0.53 (0.24–1.18)	0.60 (0.27–1.36)
Yes, with pulmonary disease^e^	6	8.8	46	13.6	0.49 (0.19–1.28)	0.62 (0.22–1.72)
						
*Nadir CD4+ cell count, cells per μl*						
⩾200	31	45.6	142	42.4	1	1
50–199	26	38.2	124	37.0	0.96 (0.54–1.71)	1.07 (0.57–2.02)
<50	11	16.2	69	20.6	0.73 (0.34–1.55)	0.87 (0.39–1.90)
Unknown	0		2			

Abbreviations: cART=combined antiretroviral therapy; CI=confidence interval; OR=odds ratio.

a See the ‘Materials and Methods’ section for definition of reference date.

b Conditioned upon matching variables.

c Conditioned upon matching variables and adjusted for smoking status (never/former, current with <30 pack-years, current with ⩾30 pack-years, unknown).

d Current smokers only.

e Includes recurrent bacterial pneumonia, pulmonary tuberculosis, or *Pneumocystis carinii* pneumonia.

**Table 3:** Relative risk of lung cancer, by markers of immunodeficiency at two different time periods before cancer diagnosis

	**One to two years before lung cancer^a^**						**Within one year before lung cancer^a^**					
	**Lung cancer**		**Controls**				**Lung cancer**		**Controls**			
	***N***	**%**	***N***	**%**	**OR^b^ (95% CI)**	**Smoking-adjusted OR^c^** **(95% CI)**	***N***	**%**	***N***	**%**	**OR^b^ (95% CI)**	**Smoking-adjusted OR^c^ (95% CI)**
Overall	68		337				68		337			
												
*CD4+ cell count, cells per μl*												
⩾500	23	39.0	100	34.2	1	1	20	29.4	119	36.4	1	1
200–499	26	44.1	146	50.0	0.76 (0.41–1.43)	0.59 (0.30–1.16)	38	55.9	159	48.6	1.41 (0.78–2.54)	1.11 (0.59–2.10)
<200	10	16.9	46	15.8	0.96 (0.41–2.24)	0.97 (0.40–2.34)	10	14.7	49	15.0	1.21 (0.49–2.96)	1.19 (0.47–3.04)
Unknown	9		45				0		10			
												
*CD8+ cell count, cells per μl*												
⩾1000	28	47.5	122	41.9	1	1	26	38.2	132	40.4	1	1
500–999	25	42.4	128	44.0	0.84 (0.46–1.53)	1.05 (0.55–1.98)	35	51.5	156	47.7	1.15 (0.65–2.05)	1.11 (0.60–2.04)
<500	6	10.2	41	14.1	0.64 (0.25–1.67)	0.57 (0.20–1.58)	7	10.3	39	11.9	0.92 (0.37–2.29)	0.77 (0.30–1.97)
Unknown	9		46				0		10			
												
*CD4+/CD8+ ratio, %*												
⩾0.50	25	42.4	125	43.0	1	1	22	32.4	140	42.8	1	1
0.25–0.49	22	37.3	109	37.5	1.01 (0.53–1.90)	0.78 (0.39–1.55)	26	38.2	120	36.7	1.38 (0.74–2.57)	1.14 (0.58–2.23)
<0.25	12	20.3	57	19.6	1.07 (0.49–2.36)	0.92 (0.40–2.09)	20	29.4	67	20.5	2.15 (1.00–4.59)	2.12 (0.94–4.77)
Unknown	9		46				0		10			
												
*HIV viral load, copies per ml*												
<500	37	68.5	192	71.4	1	1	40	72.7	207	74.5	1	1
500–9999	12	22.2	36	13.4	1.79 (0.83–3.86)	2.05 (0.90–4.67)	8	14.5	37	13.3	1.15 (0.49–2.70)	1.27 (0.50–3.21)
⩾10 000	5	9.3	41	15.2	0.66 (0.24–1.79)	0.44 (0.15–1.29)	7	12.7	34	12.2	1.10 (0.44–2.75)	0.81 (0.32–2.07)
Unknown	14		68				13		59			

Abbreviations: CI=confidence interval; OR=odds ratio.

a Or before the reference date in controls (see the ‘Materials and Methods’ section for definition).

b Conditioned upon matching variables.

c Conditioned upon matching variables and adjusted for smoking (never/former, current with <30 pack-years, current with ⩾30 pack-years, unknown).

## APPENDIX

The members of the Swiss HIV Cohort Study are J Barth, M Battegay, E Bernasconi, J Böni, HC Bucher, P Bürgisser, C Burton-Jeangros, A Calmy, M Cavassini, M Egger, L Elzi, J Fehr, M Flepp, P Francioli (President of the SHCS), H Furrer (Chairman of the Clinical and Laboratory Committee), CA Fux, M Gorgievski, H Günthard (Chairman of the Scientific Board), B Hasse, HH Hirsch, B Hirschel, I Hösli, C Kahlert, L Kaiser, O Keiser, C Kind, T Klimkait, H Kovari, B Ledergerber, G Martinetti, B Martinez de Tejada, N Müller, D Nadal, G Pantaleo, A Rauch, S Regenass, M Rickenbach (Head of Data Center), C Rudin (Chairman of the Mother and Child Substudy), P Schmid, D Schultze, F Schöni-Affolter, J Schüpbach, R Speck, P Taffé, A Telenti, A Trkola, P Vernazza, V von Wyl, R Weber and S Yerly.
